# Gait Event Detection during Stair Walking Using a Rate Gyroscope

**DOI:** 10.3390/s140305470

**Published:** 2014-03-19

**Authors:** Paola Catalfamo Formento, Ruben Acevedo, Salim Ghoussayni, David Ewins

**Affiliations:** 1 Centre for Biomedical Engineering, Faculty of Engineering and Physical Sciences, University of Surrey, Guildford, Surrey, GU2 7XH, UK; E-Mails: s.ghoussayni@surrey.ac.uk (S.G.); d.ewins@surrey.ac.uk (D.E.); 2 School of Engineering, Universidad Nacional de Entre Ríos, 3101 Oro Verde, Entre Ríos, Argentina; E-Mail: racevedo@bioingenieria.edu.ar; 3 CONICET, 3101 Oro Verde, Entre Ríos, Argentina

**Keywords:** gait event detection, initial contact, foot off, gyroscopes, algorithms, ambulatory system, gait analysis, stair ascent, stair descent, electrical stimulation, foot drop

## Abstract

Gyroscopes have been proposed as sensors for ambulatory gait analysis and functional electrical stimulation systems. These applications often require detection of the initial contact (IC) of the foot with the floor and/or final contact or foot off (FO) from the floor during outdoor walking. Previous investigations have reported the use of a single gyroscope placed on the shank for detection of IC and FO on level ground and incline walking. This paper describes the evaluation of a gyroscope placed on the shank for determination of IC and FO in subjects ascending and descending a set of stairs. Performance was compared with a reference pressure measurement system. The absolute mean difference between the gyroscope and the reference was less than 45 ms for IC and better than 135 ms for FO for both activities. Detection success was over 93%. These results provide preliminary evidence supporting the use of a gyroscope for gait event detection when walking up and down stairs.

## 1. Introduction

Applications such as ambulatory systems measuring activities of daily living, or the use of Functional Electrical Stimulation (FES) systems for correction of foot drop, often require detection of the initial contact (IC) of the foot with the floor and the final contact or foot off (FO) during extended periods of outdoor walking. Using equipment normally found in gait laboratories, which are commonly used for indoor gait event detection, such as force platforms [1–3], optical systems [4–6] and pressure matrices [7–9] may not represent an appropriate option for this task.

Alternative options include the use of body-mounted inertial sensors, such as gyroscopes and accelerometers. These sensors present advantages such as portability, low cost and low current consumption that make them appropriate for long duration outdoor ambulatory monitoring. Also, they can be mounted so that they remain under the clothes, improving system cosmesis, and potentially so that no specialist footwear or footwear modifications are needed. Recently, systems including inertial sensors have been proposed for the measurement of spatio-temporal gait parameters [10,11], joint angle [12–14] and for activity classification [15]. Investigations into the most appropriate sensor arrangement continues as the number and type of sensors, and their positioning and measurement algorithms, are evaluated for different applications.

Gyroscopes placed on the shank have been proposed for ambulatory gait analysis [12,13,16–19]; for the timing [20,21] and as feedback [14] of FES systems; and for a control system for lower limb prostheses and orthoses [22]. In terms of gait event detection, a gyroscope placed at the shank has proven to be acceptably accurate in healthy [15,23,24] and pathological gait when walking on level ground [24–26] and in healthy gait walking up and down an incline [27].

Gyroscopes placed on the shank and on the foot have been proposed for gait pattern classification, including ascending and descending stairs [15,28–31], and it was concluded that it is possible to reliably determine the type of activity performed by the subjects, yet only one study has evaluated the timing performance of a gyroscope placed on the shank for detection of two gait events (heel off and heel on) during a variety of activities, including climbing stairs, with the final objective of using the gyroscope as a sensor for triggering functional electrical stimulation systems [21]. The evaluation was performed on one subject, who had an implanted electrical stimulation system, comparing data to that determined from a heel switch. However, due to false detections from the heel switch on stair walking, it was not possible to measure the difference in timing between the sensor system and the reference for that particular activity. The purpose of the work outlined in this paper was to evaluate the detection of IC and FO, using one gyroscope placed on the shank of subjects walking up and down a set of stairs, measuring the difference in detection between the proposed algorithm and a reference method.

This study is part of an investigation at the University of Surrey directed at the evaluation of gyroscopes as sensors for outdoor gait event detection and their use for FES drop foot correction systems. Previous research included the evaluation of a gyroscope placed on the foot above the metatarsals in unimpaired subjects and in subjects with foot drop [32], and the evaluation of a gyroscope placed on the shank also in unimpaired subjects and subjects with foot drop [20]. Later, a study extended the scope of application to children, with the evaluation covering both level ground and incline walking [27]. As there are notable differences in the angle of the knee at IC and FO for stair ascent, stair descent and level ground ambulation [33–35], it is possible that the detection of events using a given algorithm based on shank angular velocity may be affected. The present research expands our earlier work, evaluating the detection of these two gait events using one gyroscope placed on the shank of children when walking up and down a set of stairs, using the same algorithm implemented for level ground and incline walking.

To summarise, when used as part of an FES system for correction of foot drop, the sensor approach used for triggering and stopping stimulation should ideally be acceptably accurate and reliable in the detection of events for all possible terrains, including stairs. Changes in kinematic profiles between terrains experienced in activities of daily living may impact on the accuracy of an algorithm that was developed from tests using, for example, level ground walking only.

## 2. Methods

### 2.1. Subjects

Seven subjects (five females and two males, 8–16 years of age, 1.38–1.87 m in height and 33.3–65.3 kg in mass) without discernible gait abnormalities participated in the study. The protocol was explained to the subjects and their parents and a consent form was signed by every parent and each subject. The study was reviewed and approved by the Local Research Ethics Committee.

### 2.2. Protocol

The subjects walked at a self selected ‘normal’ speed, wearing the shoes they use routinely for daily activities. The walking circuit included outdoor level ground and stairs. The subjects walked approximately 5 m on level ground, then stopped for a few seconds, then walked up the stairs, stopped at the end of the staircase and continued for another 5 m on level ground. After a short break, they repeated the circuit, walking on level ground, stopping a few seconds, then down the stairs, stopping once again, followed by level walking again. The stairs consisted of six steps, five of which had a 250 mm tread and 150 mm height, and the final (lowest) step that led to level ground had a 250 mm tread and 10 mm height. Only the data collected on the stairs was used for the analysis. The last Initial Contact, performed on level ground, was also considered.

### 2.3. Gyroscope Data

A single axis gyroscope (ENC 03J, Murata Manufacturing Co., Ltd., Nagaokakyo-shi, Kyoto, Japan) was placed on the anterior aspect of the shank of the dominant leg, 50 to 100 mm below the tibia tuberosity, aligned with the long axis of the tibia and positioned so as to measure the angular velocity of the shank in the sagittal plane.

**Figure 1.**
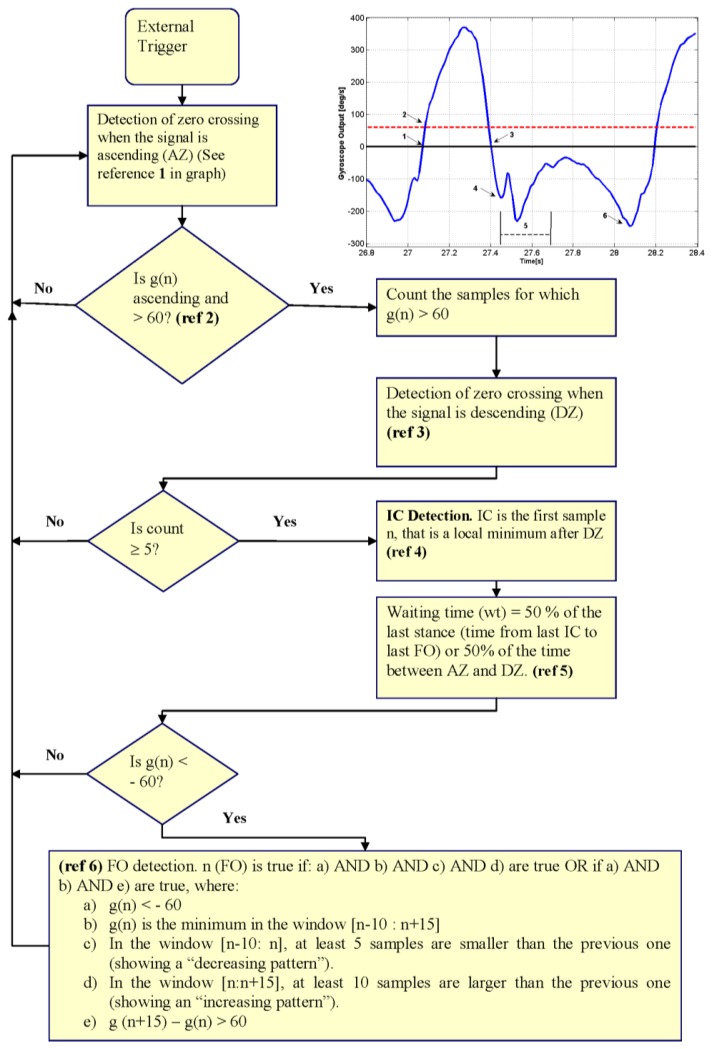
Flowchart of the algorithm used for detection of initial contact (IC) and foot off (FO) from the gyroscope signal (adapted from [27]). The graph corresponds to data collected from one subject who participated in the study. g(n) is the value of the gyroscope signal at the sample n; AZ: ascending zero crossing; DZ: descending zero crossing; and wt: waiting time. The numbers in the graph relate to steps of the algorithm: (1) detection of ascending zero crossing; (2) detection of signal exceeding threshold of 60 deg/s (equivalent to a system output of 0.2 V for this configuration); (3) detection of descending zero crossing; (4) IC detection; (5) waiting time; and (6) FO detection. After FO detection, the algorithm starts again.

A rule-based algorithm that evaluates each sample sequentially, starting from the onset of the external trigger was used for detection of events. The algorithm was described in detail in [27]. Figure 1 shows the flowchart of the algorithm and a typical gyroscope signal from one of the subjects. The rules for the algorithm as well as the parameters chosen were determined empirically using preliminary data from two subjects walking on level ground and incline as described before [27]. The rules and parameters were not changed for the present study.

The determination of IC and FO events is based on the detection of two negative peaks in the shank angular velocity signal. Initially, the algorithm searches for the swing phase of the cycle using the null output and a threshold (References 1–3 in Figure 1). The first negative minimum after swing is defined as IC (Reference 4 in Figure 1). After IC, a waiting time is set (Reference 5 in Figure 1). The first negative peak after the waiting time is over is used to determine the FO (Reference 6 in Figure 1). Once FO has been determined the algorithm starts again, by looking for the following positive wave (swing phase of gait).

### 2.4. Reference System

As a reference system, the FScan Mobile System was used. The system has been shown to have acceptable accuracy for detection of IC and FO when using a Contact Area method [36]. The data sources were synchronized using an external switch and data were sampled at 125 Hz.

### 2.5. Data Analysis

The data analysis was performed following the same protocol as previous work [27]. Once the events were determined for each method, the comparison was performed by calculating the differences in time between them for the detection for each step analysed. The differences in timing between the Contact Area (CA) and the Gyroscope (GD) detection algorithms were calculated by subtracting the time of GD detection from that of CA detection. Also, the absolute mean difference (AMD) for each step was calculated. The differences and the AMD for all the steps for each subject were then averaged so that a single value was obtained for each subject and each condition. In order to compare the results with previously reported data, the mean differences for all the subjects were averaged and reported together with the 95% confidence interval (CI) [37]. The relatively small number of events detected per subject was considered adequate for a pilot evaluation of the algorithm, however, they were insufficient to perform a robust statistical analysis of the differences.

In addition, the distributions of the differences were plotted in histogram form. For each event, the number of events *versus* the time difference expressed in ms (calculated in the range between −400 to 400 ms, divided in 10 ms intervals) was calculated. Positive differences indicate that the GD method detected the event earlier than CA.

Finally, the success in detection was calculated. An error in detection was defined as an event missed or wrongly detected (as an extra event in a gait cycle for which another, correct, event had been visually detected). Then, the “success in detection” was calculated as the total number of events correctly detected by the gyroscope divided by the total events detected by the reference method and multiplied by 100.

## 3. Results and Discussion

**Figure 2.**
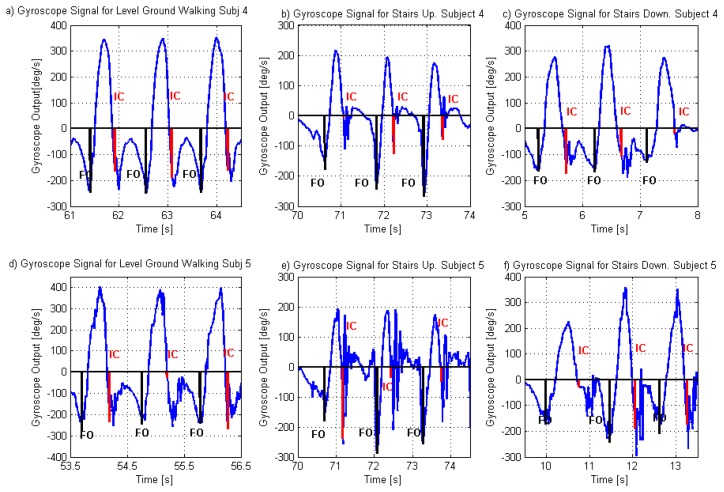
Gyroscope signals from Subject 4 (**a**–**c**) and Subject 5 (**d**–**f**). Vertical lines in the gyroscope graphs indicate the characteristic features of the gyroscope signal used for the detection of IC and FO events. (**a**) and (**d**) are the gyroscope signals for the subjects walking on level ground; (**b**) and (**e**) corresponds to walking up the stairs; and (**c**) and (**f**) corresponds to walking down the stairs.

Seven children completed the stair descent trial, with a total of 31 IC and 27 FO, while six children completed the stair ascent trial, with a total of 24 IC and 20 FO. Figure 2 shows gyroscope signals for two of the subjects who participated in this study, with the corresponding IC and FO detection. These example signals were selected as they represent the extreme signal patterns from this group. The gyroscope signals during stair walking for Subject 4 were the most similar to the level ground pattern while the signals for Subject 5 were the most different. Figure 2a,d shows the signals for Subjects 4 and 5 respectively while walking on level ground (both corresponding to the path before the stair walking). The signals for level ground walking present a clear pattern, including positive shank angular velocity during the swing phase and negative velocity during the stance phase of gait (for this project, the null output of the circuit was measured before starting each walk and this value was used as the zero).

Walking down the stairs present a similar pattern for the signals for both subjects (Figure 2c,f). However, the signal changes with a less ‘smooth’ behaviour during the stance phase. The main differences in the signal pattern, however, are when subjects walked up the stairs. In this case, for Subject 4 (Figure 2b), there is still a clear pattern for the swing and stance phases, although the velocity of the shank reaches positive values during stance (indicating a temporal counterclockwise movement of the shank), which was also noted in the gyroscope signal by other researchers [31] and in the movement of the leg by other authors [38]. In particular McFadyen and Winter [38] observed that for walking up the stairs, during a phase that they called “pull up” phase (which extended from beginning of single leg support to approximately mid swing of the contralateral leg) the leg moved backwards, which increased the vertical position of the knee and, in conjunction with knee extension, provided lift to the body. The gyroscope signal for Subject 5, has a less regular pattern, with more oscillations during the stance phase and less clear differentiation between swing and stance phase. Figure 2d shows that the gyroscope signal for Subject 5 also had oscillations during the stance phase of level ground gait. These oscillations are common around the time of IC and briefly afterwards and may be related to events occurring during the loading response. The impact produced when the foot hits the floor at the time of IC may cause artefact movements to the sensor; in which case, the amplitude of the oscillations may be greater if the sensor is not tightly attached to the shank. Due to the presence of the oscillations, the detection algorithm includes a waiting time after IC was detected and before the search for the FO starts (Reference 5 in Figure 1). The oscillations during the stance phase of stair ascent gait are more pronounced than during level ground walking but the reason for the increase remain unknown. Kinematic data from an optoelectronic system could provide further information regarding the actual movement of the shank that could be used to identify the causes.

For all the subjects there was a clear reduction of the positive velocity during the swing phase of gait during stair ascent.

### 3.1. Mean Differences and Distribution of the Differences

The absolute mean difference (AMD), the mean difference (MD) and 95% confidence interval (CI) (expressed in ms) in the detection of initial contact (IC) and foot off (FO) between the gyroscope and the reference system for walking up and down the stairs are shown in Table 1. Also, in order to compare the results with previously reported results on level ground and walking on an incline, the differences obtained for these terrains are included in Table 1.

**Figure 3.**
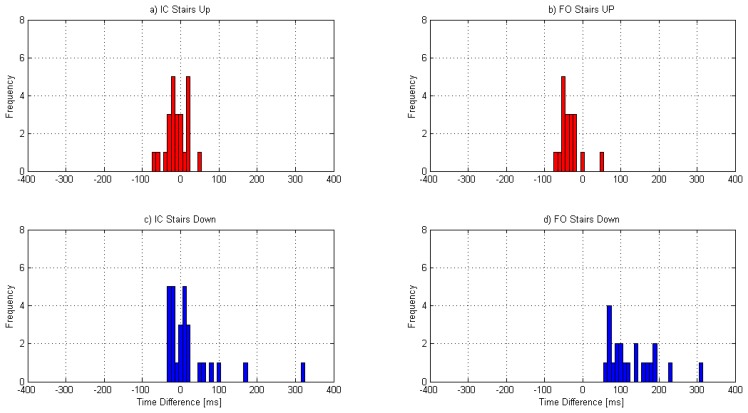
Frequency distribution of the time differences between the gyroscope and the reference method for IC and FO event detection for up the stairs walking (**a**) and (**b**); and down the stairs walking (**c**) and (**d**). The number of events considered was 24 IC and 18 FO for Stairs Up and 28 IC and 21 FO for Stairs Down. Positive differences indicate that the gyroscope method detected the event earlier than the reference.

Figure 3 shows the histograms of the time differences in IC and FO detection for the up stairs and down stairs walking. In order to avoid any bias in the histograms due to the different number of events detected for each subject, the maximum number of steps available for all subjects was considered. Hence four IC events and three FO events were considered for each child for each condition.

Figure 3a,b show the histograms of the differences between the methods for the IC and FO event, respectively, during walking up the stairs. For this trial, 24 IC and 18 FO events were considered. Figure 3c,d show the histograms of the differences for walking down the stairs, for which 28 IC and 21 FO were considered.

From Table 1, it can be seen that the AMD for walking up the stairs is of the same order as that for level ground and incline walking for both events. However, the AMD between the gyroscope and the reference is larger for walking down the stairs, especially for detection of FO.

The MD in the detection of IC on stairs is similar to other terrains and although the MD in the detection of IC while walking down the stairs is positive (indicating that the gyroscope detected the event earlier than the reference) unlike what happened with the other terrains, the histogram (Figure 3c) indicates that most of the events are distributed around zero.

FO detection changed on stairs walking with respect to the other terrains. For up stairs, GD detected FO generally later than CA (Figure 3b), unlike the detection on level ground, incline and down the stairs walking [27]. This change in the pattern may be explained by the characteristic pattern of the leg movement while walking up the stairs as described by McFayden and Winter [38]. These authors described the first part of the swing as a phase that involves not only bringing the leg up and over to the next step, but also keeping the foot clear of the intermediate step. According to the authors, this was accomplished in two ways: tibialis anterior contracting in order to provide sufficient dorsiflexion and the leg being pulled back through flexion at the knee. This backwards movement of the leg and flexion of the knee is more pronounced than that for level walking and several authors agree that the extension of the knee (and consequent anticlockwise movement of the shank) is delayed from FO with respect to level ground walking [33,34,38]. The feature (negative peak) from angular velocity that was used in this study for FO detection (Reference 6 in Figure 1) represents the time when the movement backwards of the shank slows down to change into a movement in the opposite (forward) direction. If the flexion of the knee is prolonged and the extension movement is delayed, then it is possible that this peak is also delayed. This could be an explanation for the delay in detection of the FO of the GD with respect to CA in the detection for up the stairs walking.

For down the stairs walking, GD detected FO earlier than CA (see Figure 3d), which coincides with previous results on level ground and incline walking [27], but the differences are larger. Riener *et al.* [34] showed that the extension of the knee starts earlier in stair descent than on level ground walking and McFadyen and Winter [38] also noted that the knee flexion during early swing was only slight as foot clearance was not as imperative. Following a similar reasoning as for up the stairs, it is possible that the negative peak in the angular velocity that is used to detect FO (Reference 6 in Figure 1) could happen earlier in stair descent than in level ground walking and this could explain the larger differences seen in the detection of FO by GD with respect to CA for stair descent.

There is no comparative data for detection on stairs as there is only one paper that addressed it and due to technical issues timing data for stairs was not reported. Still, the range of results for this study are comparable to those found by other investigators who proposed detection algorithms using single gyroscopes placed on the shank, even when the evaluations were not performed on stairs and the reference system used varies across studies. Lee and Park [19] reported the results of an algorithm evaluated in five healthy adults walking at three self-selected walking speeds by comparing its performance against four footswitches placed on sole of the foot. Averaged measurements over the three walking speeds showed that the algorithm detected the FO events earlier (−8 ms on average with the 95% confidence interval of [−11, −6] ms) and the ICs later (19 ms with the interval of [17, 21] ms) than the footswitch-based method. Kotiadis *et al.* [21] evaluated an algorithm on a subject implanted with a drop foot stimulator and compared their performance with a foot switch, while the subject walked on different terrains. The reported MD varied from 10 ms (for carpet walking) to −35 ms (for rough terrain) for heel down and from −60 to −90 ms for heel off (their results were calculated so that negative differences corresponded to events detected later than the reference). The distribution of the differences varied across these studies, possibly due to the reference system used.

The reference system used for this study (FScan insoles) showed an absolute mean difference in event detection with respect to a gold standard (force platforms) of 22 ms for IC and 10 ms for FO. So the actual differences between gyroscope detection and the gold standard could be larger than the ones reported here (i.e., absolute mean differences could be 22 ms larger for IC and 10 ms larger for FO).

### 3.2. Reliability

In terms of the success in detection, the total number of events detected during stair ascent was 44 (24 of which were IC and 20 FO). Of these, the gyroscope missed two FO events, which means that the success in detection was 95.5%. The total number of events detected during stair descent was 58 (31 IC events and 27 FO events), of which the gyroscope missed three FO and detected wrongly one FO, so the success in detection was 93.1%.

**Figure 4.**
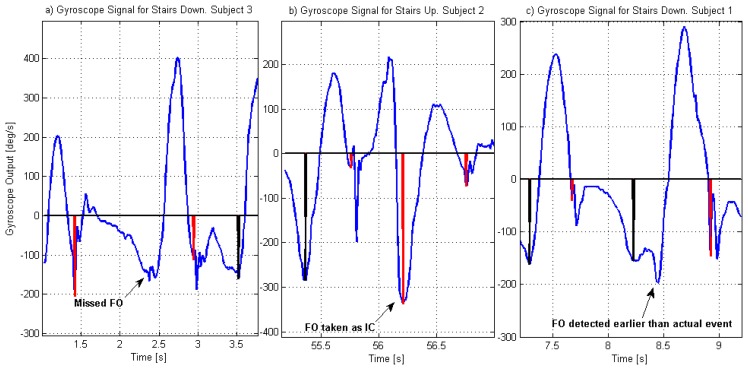
Causes for missed or wrongly detected FO events. Gyroscope signals from Subject 3 (**a**), Subject 2 (**b**) and Subject 1 (**c**). Vertical lines in the gyroscope graphs indicate the characteristic features of the gyroscope signal used for the detection of IC and FO events. (a) FO event missed since it did not comply with condition of local minimum; (b) FO event wrongly detected as IC event due to the characteristics of stance phase data; (c) FO event detected earlier than the actual event, as the signal at that point complied with the conditions imposed for local minimums.

The errors in detection have three causes. Three FO events missed (all of them during stair descent) presented themselves as an area of local minimums, rather than a single negative peak (see Figure 4a). In those cases, although the signal reached the threshold of –60 deg/s and the waiting time was appropriate, none of the possible points complied with the condition of being the minimum in a window of 26 samples (condition b, reference 6 in Figure 1). Another two FO events were missed (both while walking upstairs) and wrongly detected as IC, as the gyroscope signal during the stance phase of gait had similar amplitude and duration characteristics to the swing phase (see Figure 4b). In these cases, the previous and subsequent IC events were correctly detected, but the detection of FO failed. Finally, one FO event was detected earlier than the actual event happened, given that there was a local minimum that complied with all the requirements needed for a FO.

The selection of an appropriate window (in this case it was selected as 26 samples or 200 ms) to identify the FO event represents a compromise. A smaller window may improve the sensitivity to detect a local minimum, avoiding errors such as the one seen in Figure 4a. However, it increases the probability of detecting spurious peaks that are not real events, such as the one seen in Figure 4c. A larger window could avoid false detections but it would diminish the sensitivity of the algorithm.

Further work is required to investigate the causes of the changes in the signal, for example do they reflect infrequent but ‘real’ changes in movement patterns, e.g., resulting from instability in the foot positioning when walking down the stairs, or are they artefacts resulting from poor attachment of the sensor to the body; our view is that the former is more likely.

Implementation of the current algorithm as part of an FES stimulator could include rules to diminish the effect of the errors. For example, in the cases shown in Figure 4a,b, once the waiting time is over, if the signal has reached and went below −60 deg/s, and it is now above this and FO has not been detected, then the stimulation should start. However the effect of this delay needs evaluation, especially for walking up the stairs for which the clearance of the foot is challenged.

**Figure 5.**
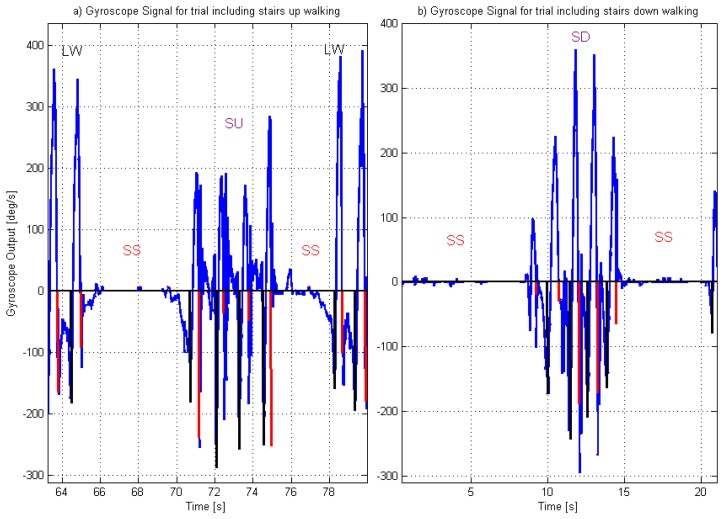
Gyroscope signal for subject 5, for the trial including ascending stairs (**a**) and the one including descending stairs (**b**). Vertical lines indicate the detected events. Subjects walked on level ground walking (LW), then stopped and stood still (SS) before and after walking up (SU) or down (SD) the stairs.

The walking circuit included outdoor level ground and stairs. The subjects walked approximately 5 m on level ground, then stopped for a few seconds and then walked up the stairs, stopped for a few seconds and continued for another 5 m on level ground. After a short break, they repeated the circuit, walking on level ground, stopping for a few seconds and then down the stairs, stopping and followed by level walking again. Figure 5 shows the gyroscope signal while subjects walked on stairs, while they were standing still and while walking on level walking. Only the data collected on the stairs was used for the analysis, of which the first step was always detected after the person had stopped. It is possible to see from the figure that the algorithm did not detect events during small movement of the leg during standing still. It may be that movements associated to weight shift or shuffling do not imply important movement and orientation change of the shank [21].

The algorithm requires a first swing phase (*i.e.*, a forward movement of the leg, performed at a requested velocity of 60 deg/s, during a stated time of at least five samples or 40 ms) to start detection. Small movements of the leg do not comply with these requirements, hence it is not until a proper swing of the leg happens that the algorithm starts detecting events. Once a swing phase has occurred, with or without information from the previous step, the algorithm starts or continues with detection.

If the algorithm has started, then the person stops and then starts walking (as seen in Figure 5a) then the first FO after standing still will be detected. If, the algorithm starts while the person is standing still (as shown in Figure 5b) then it will wait to detect a swing phase, hence the first IC will be detected. From the rules of the algorithm, and based on the results from our experiments, there is no reason to believe that stopping during stairs will affect the algorithm output.

The movement of reversing the walk direction (for example at the end of the stair case or in the middle of the stairs) has not been tested and could be included in future evaluations of the algorithm. However, taking into account that large angular velocities are needed prior to detection of events, reversing is not expected to influence the event detection.

The stair walking proved to be the most challenging terrain for the algorithm given that the same algorithm detected 99.5% of events on level ground and incline walking [27]. This was partly due to the change in signal presentation during stair walking, as described previously.

Kotiadis *et al.* arrived at similar results [21]. The algorithm using one gyroscope on the shank showed 100% correct detection on level ground (including carpet and rough terrain) and stairs ascent. However, for stairs descent, of 11 actual steps the algorithm detected correctly only five and detected nine false events.

### 3.3. Final Considerations

Different algorithms have been proposed in the literature for detection of gait events using gyroscope data, with rule-based algorithms being the most common [19,21,24,26]. Also more sophisticated algorithms that included wavelet analysis [23,25], weighted-frequency Fourier linear combiner (WFLC) [39] and Hidden Markov Models [40] have been proposed. The performance of the algorithms is still under evaluation, while their accuracy and reliability are been assessed for different pathological conditions and terrains. While it has been stated that the most important requirement for a successful use in an ambulatory rehabilitation system is sufficient reliability in the detection of gait events during daily use, it has also been acknowledged that the challenge and most difficult task in the future design of these systems is the proper interpretation of the measured signals by an algorithm with low demands for CPU capacity and memory [41]. Rule-based algorithms are less computationally demanding than most complex ones. It has also been reported that a rule-based algorithm performed nine times faster than an algorithm based on wavelets analysis [26], which represents an advantage for on-line systems.

The algorithm proposed in this study is rule-based and evaluates sequentially each sample, which facilitates the conversion into an on-line algorithm. However, the detection of FO requires the analysis of 15 samples ahead of the sample being evaluated. Consequently, if the algorithm were to be directly converted to an on-line process, the FO detection would have a delay of approximately 120 ms. If this delay was unacceptable, further rules could be added (for example, the use of specific parameters for each subject such as a threshold on the gyroscope signal for FO detection).

Once a sensor system has been proven to be accurate and reliable for detection of gait events on different terrains for subjects with and without stimulation, it will be possible to explore different stimulation strategies for different terrains. Several algorithms using body-worn sensors have been proposed for activity classification, including decision trees or hidden Markov models (a review of classification techniques, describing the advantages and disadvantages for each approach can be found in [42]). The algorithm proposed in this study does not include activity identification. However, as gyroscopes placed on the shank on their own [31] or combined with accelerometers [43] have proven reliable for classifying walking conditions, it is reasonable to expect that combined algorithms could be implemented to achieve activity classification and event detection, if the application requires it.

This is a preliminary study that intended to evaluate the detection of IC and FO, using one gyroscope placed on the shank of subjects walking up and down a set of stairs. Considering the differences reported in the angle of the knee at IC and FO for stair ascent, stair descent and level ground ambulation [33–35], it was possible that the detection of events using a given algorithm based on shank angular velocity may be affected. Based on the promising results obtained in this study, event detection through a gyroscope mounted on the shank should be evaluated on subjects with pathological gait, especially those candidates for using an FES system for foot drop or toe walking correction. Ideally the evaluation should be performed under two conditions: with and without stimulation. Previous work at University of Surrey [20] supports the evaluation of the gyroscope in patients presenting with foot drop and toe walking.

## 4. Conclusions

Practical systems capable of accurate detection of gait events would be useful in many ambulatory applications. Gyroscopes placed on the shank have been proposed as part of ambulatory gait analysis and FES systems. We have reported earlier on evaluation of an algorithm for a single gyroscope placed on the shank for level ground and incline walking. This work reports on the results of the evaluation for stair ascent and descent. Although this is a preliminary study with a relatively small sample size, the results (absolute mean differences smaller than 45 ms for IC and better than 135 ms for FO, and overall reliability: better than 93%) are comparable to previous ones reported in the literature for other event detection algorithms using gyroscopes on the shank. Based on the results obtained, the authors believe that event detection through a gyroscope mounted on the shank is worthy of further study. Future work should include evaluation of the sensor on subjects presenting with pathological gait, especially patients who use FES systems for foot drop or toe walking correction. The evaluation should be performed under both conditions: with and without stimulation.

## Figures and Tables

**Table 1.** Absolute mean difference (AMD) ± one standard deviation, Mean difference (MD) ± one standard deviation and 95% Confidence Interval (CI), all expressed in milliseconds, in the detection of Initial Contact (IC) and Foot Off (FO) between the gyroscope and the reference system for Up Stairs walking (US) and Down Stairs walking (DS). Positive MD indicates that the gyroscope method detected the event earlier than reference. n = 7 for walking up the stairs, and n = 6 for walking down the stairs. Also, data from a previous study for Level Ground Walking (LG), Incline Up (IU) and Incline Down (ID) walking are included [27]. (Results from the previous study are shown with a *)

	**AMD (ms)**				**MD (ms)**				**CI (ms)**		
		IC	FO		IC		FO	IC		FO
**US**		**21±17**	**40 ± 13**		**−11 ± 18**		**−35 ± 20**	**[−30; 9]**		**[−56; −14]**
**DS**		**38 ± 36**	**132 ± 44**	**18 ± 46**		**132 ± 44**		**[−25; 61]**		**[92; 173]**
LG*		15 ± 6	50 ± 14		−8 ± 9		50 ± 14	[–16; 1]		[37; 63]
IU*		24 ± 12	43 ± 10		–21 ± 15		43 ± 10	[–35; –8]		[34; 52]
ID*		20 ± 11	73 ± 12		–9 ± 20		73 ± 12	[−29;12]		[60; 85]
